# Impact of a one-year interruption to vector control on Bioko Island, Equatorial Guinea

**DOI:** 10.1101/2025.11.04.25339542

**Published:** 2025-11-06

**Authors:** David S. Galick, Dianna E.B. Hergott, Norberto Bosepa Cubacuba, Teresa Ayingono Ondo Mifumu, Jordan M. Smith, Matilde Riloha Rivas, Wonder P. Phiri, David L. Smith, Carlos A. Guerra, Guillermo A. García

**Affiliations:** 1 MCD Global Health, Malabo, Equatorial Guinea; 2 Institute for Health Metrics and Evaluation, University of Washington, Seattle, WA, USA; 3 Ministry of Health and Social Welfare, Malabo, Equatorial Guinea; 4 MCD Global Health, Silver Spring, MD, USA

## Abstract

Over the last 20 years, malaria transmission on Bioko Island, Equatorial Guinea has declined dramatically thanks to the implementation of robust malaria control activities, centered around island-wide indoor residual spraying (IRS). In 2024 Bioko Island experienced a lapse in malaria control funding, and as a result vector control activities (including IRS) were interrupted. However, agreements with funders allowed for both a previously planned malaria indicator survey (MIS) and the subsequent implementation of indoor residual spraying (IRS) in late 2024. This study analyses routine case data from public health facilities from 2019–2024 and annual cross-sectional MIS data from 2019–2024 using interrupted time series methods to quantify the impact of the interruption and reestablishment of control activities on Bioko Island. In 2024, the number of confirmed cases reported was 41% higher than the 2021–2023 average, and the *Plasmodium falciparum* prevalence rate (*Pf*PR) rose by three percentage points. Statistical modeling estimated that 25.3% (95% CI 12.0–36.3%) of 2024 cases were avertable if control activities had been maintained, and that the interruption was associated with an increased *Pf*PR in 2024, above previous trends (adjusted OR 1.16, 95% CI 1.05–1.29). Moreover, the reintroduction in IRS in late 2024 was found to have averted an estimated additional 7.0% (95% CI 3.0–10.2%) increase in confirmed cases. In just one year with interruptions to control, malaria transmission and burden quickly resurged on Bioko Island. However, Bioko’s experience demonstrates that the reestablishment of control activities can equally rapidly contain, and reverse resurgence associated with control interruptions.

## Introduction

Since 2000, a renewed commitment to malaria control has substantially reduced the number of cases and deaths caused by malaria worldwide, and especially in sub-Saharan Africa [1]. These gains were achieved primarily through intensive, widespread vector control activities, including both the distribution of long-lasting insecticide-treated nets (LLINs) and administration of indoor residual spraying (IRS) [2]. However, since 2015, progress has stalled, in part due to funding gaps, challenges to the effectiveness of existing tools, and a lack of new, scalable tools currently available for programmatic use [3,4]. These challenges serve as a reminder of the vulnerability of progress already made, especially in the face of interruptions to control activities.

The impacts of malaria control interruptions have long been clear. Historically, funding disruptions have been the most common cause of malaria resurgence across world regions [5]. In recent years, re-allocation of funding and external shocks such as the COVID-19 pandemic have demonstrated again the fragility of reductions in malaria transmission and burden to even partial disruptions of control activities [6–8]. Multiple modeling exercises have also explored and estimated the enormous additional burden that large interruptions in malaria control activities could cause [9–11].

Despite the overwhelming evidence that funding reductions on malaria control in areas with ongoing transmission will result in significant resurgence, the funding environment remains difficult due to political, economic, and other concerns. Given this situation, it is crucial to measure and document the effects of funding reductions when they occur, where possible. The results from such a funding disruption on Bioko Island, Equatorial Guinea in 2024 are reported here. Thanks to the continued reporting of routine data in the National Health Information System, and re-establishment of funding in late 2024 in time to conduct a malaria indicator survey (MIS) as previously planned, quantification of the impact of the interruption using interrupted time-series methods on both malaria cases and prevalence was possible.

## Methods

### Study area

Bioko Island is the largest island of Equatorial Guinea, located in the Bight of Biafra off the coast of Cameroon. The island’s population is approximately 270,000, predominantly concentrated in the capital city, Malabo [12,13]. Bioko has historically high and perennial malaria transmission, but the implementation of a robust malaria control project based on island-wide indoor residual spraying (IRS) in 2004 dramatically reduced malaria transmission and burden [14,15]. Since 2015, vector control strategies have changed: from 2015–2020 IRS was targeted to only high transmission areas, supported by island-wide long-lasting insecticide treated net (LLIN) mass distribution campaigns in 2015 and 2018, while since 2021 LLIN distribution has been conducted primarily via continuous distribution channels and IRS re-expanded to target the entire island at varied coverage levels [16,17]. In 2022–2023, the vector control portfolio also incorporated increasingly larger scale larval source management (LSM) activities, particularly in the greater Malabo area. These vector contol interventions have been supported by case management activities (including providing free diagnosis and treatment of malaria in public health facilities) and social behavioral change communication activities.

In 2024, because of delays in funding negotiations, there was an interruption of most malaria control activities. Planned activities, such as the implementation of IRS and operation of continuous LLIN distribution points were delayed or canceled. Only routine activities, such as the diagnosis and treatment of cases, and distribution of LLINs to pregnant women during antenatal care, were able to continue. This interruption continued until July, when an agreement was reached with funders to support the implementation of key malaria prevention activities, including both IRS and LLIN continuous distribution. Funders also agreed to support implementation of the annual malaria indicator survey (MIS) as originally planned, in August-September, in order to better quantify the impact of the interruption. As a result, in August, implementation of the MIS began, while IRS was conducted from late September through December. Due to a shortage of insecticide on hand, not all areas of the island could be targeted, so some with historically low transmission and high housing quality were excluded. In all targeted communities, IRS implementation followed the completion of MIS data collection.

### Routine health data

Public health facilities on Bioko report routine indicators, including confirmed malaria cases (confirmed by RDT or microscopy), to the national malaria control program (NMCP), which are subsequently digitized into a DHIS2 system. For data reported prior to October 2023, individual-level records were digitized using a Tracker module, but due to the large workload of data digitization, the NMCP implemented an aggregate data collection module starting in October 2023, which enabled much more rapid data entry while still providing key indicators. Here, aggregated monthly reported confirmed malaria case data for each of the four districts on Bioko Island (Malabo, Baney, Luba, and Riaba) from January 2021 to December 2024 were extracted for analysis on 12 June 2025. These data include reported cases from all public health facilities on Bioko Island active during the corresponding month and did not contain any identifiable information. While malaria-attributable hospitalizations and deaths are also collected, these data were not analyzed due to the relatively low number reported and potential changes in definitions used during the study period.

An important change in the health system on Bioko Island occurred during the study period: a new public hospital, the General Hospital of Sampaka, opened for services in January 2024. During the first six months of 2024, the number of patients attended in Sampaka slowly grew but remained relatively small. In the second half of the year, however, the volume of patients significantly increased after pediatric services were transferred from the Regional Hospital of Malabo (the main public tertiary facility on Bioko) to be provided instead in Sampaka.

### Malaria indicator surveys

The methodology of the annual Bioko Island MIS has been published elsewhere [18]. In brief, the questionnaire was adapted from the RBM MIS toolkit [19], and a stratified cluster sampling design based on 109 primary sampling units (PSUs) covering all inhabited areas of Bioko was used. Strata were defined according to the population density and residual local transmission as modeled in 2018 [20], and target sample sizes were set at 25% of inhabited households for PSUs in the rural/high transmission stratum, and 5% of inhabited households for PSUs in the urban/low transmission stratum for each year. The sampling frame was updated each year based on a household census conducted during an annual round of IRS (conducted between February and July), and survey interviews were conducted from August to early October each year. All consenting household members (regardless of age) were tested for malaria using a rapid diagnostic test (STANDARD Q Malaria Pf/Pan RDT, SD BIOSENSOR). Covariates used from the MIS in this analysis included self-reported LLIN use and time of house entry on the night preceding the survey interview, history of travel outside Bioko Island in the 8 weeks preceding the survey interview, and socioeconomic status (SES) based on principal components analysis of household possessions, grouped into quintiles. Note that LLIN use and house entry time questions were asked only for participants who reported sleeping at home the night preceding the survey interview, and this was the main cause of missing data in this study.

### Statistical analysis

Interrupted time series methods were used for the analysis of both routine health system and survey data. Analyses were conducted in a manner to allow for the possibility that the impacts in each district could be distinct. District-level estimates were then aggregated to compute island-wide estimates and confidence intervals.

For analysis of reported confirmed cases, a negative binomial generalized additive model with log link was fit to case data, incorporating a cyclic seasonality term and long-term trend as splines in addition to a linear term for the interruption of services in 2024. To assess the possible impact of the reestablishment of indoor residual spraying (IRS) in late 2024, cumulative achieved 2024 IRS coverage was also included as a covariate, calculated as the proportion of inhabited houses sprayed by the beginning of each month. This covariate was extracted from IRS campaign data at district level and overall, leveraging the underlying spatial decision support system which has been described in detail elsewhere [21]. All effects and the intercept were modeled as interactions with district to allow for variation in pre-interruption trends, the impacts of the interruption and re-establishment of control. Fitted models were used to estimate the number of confirmed cases under two counterfactual scenarios: 1) that there was no interruption of services in 2024 (CF1), and 2) that services were interrupted in 2024 and IRS was not reestablished (CF2). These scenarios were then used to estimate avertable cases occurring in 2024 (i.e. averted in CF1) and cases averted by the reintroduction of IRS (i.e. cases averted with respect to CF2). Confidence intervals of scenarios and avertable cases were calculated as the 95% credible interval based on a multivariate normal approximation of the posterior distribution of model coefficients [22]. No statistical adjustment for possible confounders was made, given the difficulty of merging such data with monthly routinely reported data.

Analysis of survey data followed a method which has been previously used on Bioko MIS data to analyze the impact of travel [23]. This consisted of fitting an interrupted time series logistic regression model to *Plasmodium falciparum* prevalence (*Pf*PR) data from 2019–2024, where the impact of the interruption of services was defined as the change observed in relation to the linear 2019–2023 trend. Results are reported as adjusted and unadjusted odds ratios (OR), where variables used in adjustment were recent history of travel (in the past 8 weeks), LLIN use, early house entry (before 7PM) and SES quintile, modeled as categorical covariates (binary in all cases except SES quintile). Individuals with missing covariates were excluded for both the adjusted and unadjusted analysis. The impact of interruptions, temporal trend and intercept were fit via interaction terms to allow variation by district (for both adjusted and unadjusted estimates), given the likelihood of differing trends and effects. Throughout, survey sampling weights were used to ensure estimates are representative of Bioko Island.

### Ethics approval

The protocol for the annual Malaria Indicator Survey was approved by the Technical and Ethics Committee of the Ministry of Health and Social Welfare of Equatorial Guinea and the Ethics Committee of the London School of Hygiene and Tropical Medicine (approval number 5556). All adult survey participants provided written informed consent for themselves and, where relevant, on behalf of children under 18 years of age in their household.

## Results

### Changes in confirmed cases

**Figure 1:**
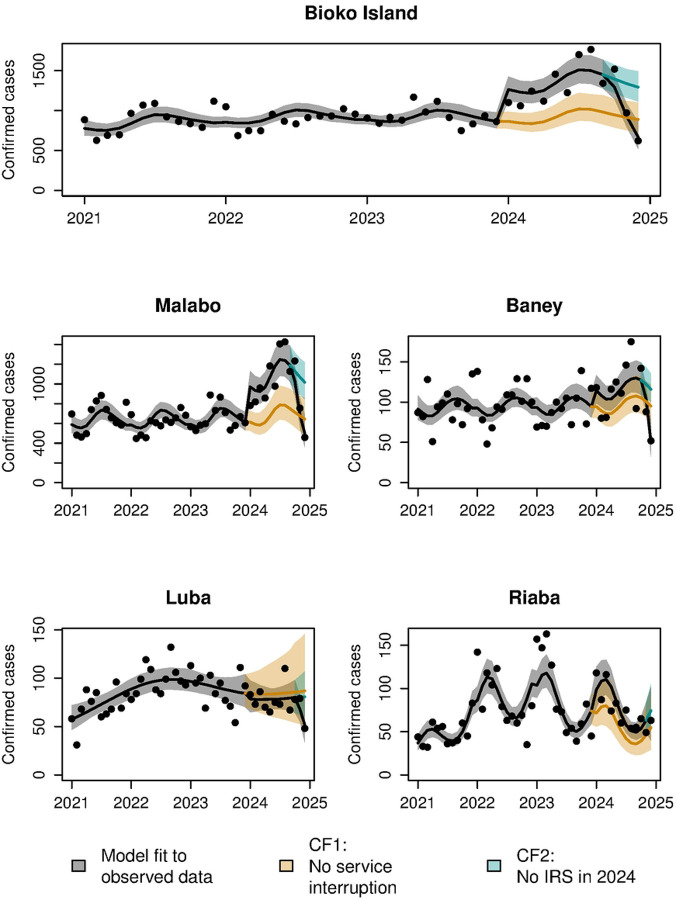
Monthly confirmed cases, fitted model and counterfactual scenarios. Points indicate the number of confirmed cases reported, while the black, blue and orange lines show the fitted model, and counterfactual scenarios CF1 (no interruption of services in 2024) and CF2 (no reestablishment of IRS in 2024), respectively. 95% confidence intervals are shown as shaded bands around lines.

**Figure 2:**
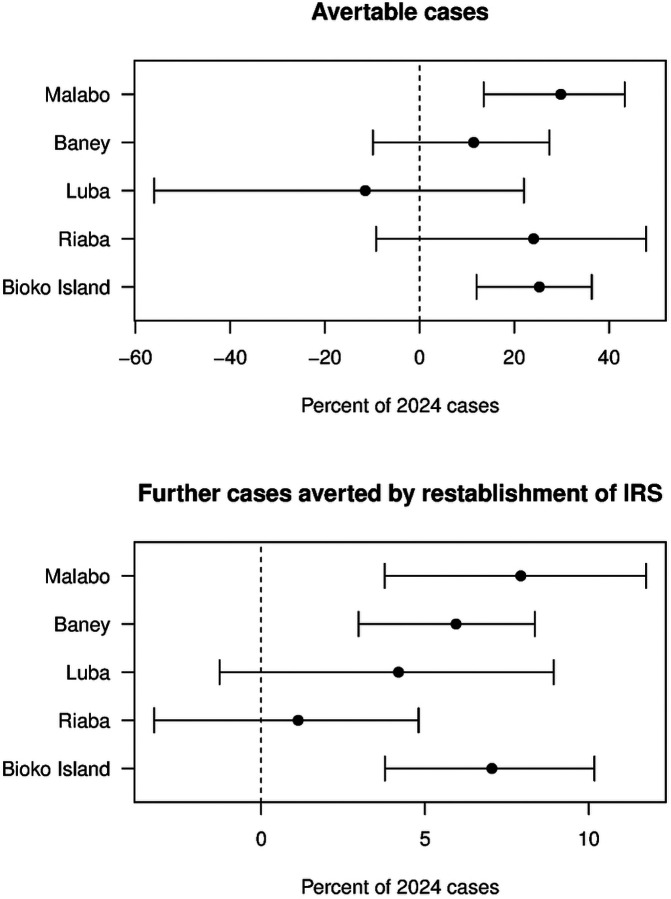
Estimated avertable cases and cases averted by reintroduction of IRS, by district. Avertable cases were calculated as the number of cases in 2024 which could have been averted if malaria control activities had not been interrupted, and further cases averted were calculated as the number of cases which would have occurred during the study period had IRS not been reestablished in late 2024. In both cases, these numbers are standardized as the percentage of predicted 2024 cases. Points show mean and whiskers indicate the 95% confidence interval.

Health facilities on Bioko reported a total of 15,121 confirmed malaria cases in 2024, which comprised an increase of 41% compared to the 2021–2023 average (10,761 per year). There was also a precipitous decline in cases in the final months of 2024, corresponding to the period after the reintroduction of IRS. Statistical modeling of counterfactual scenarios showed that the observed increase in cases in 2024 was unexplained by previous trends, while the decline in cases from October 2024 onwards was associated with achieved IRS coverage (Figure 1). Comparison of the number of predicted cases across scenarios showed that overall, 25.3% (95% CI 12.0–36.3%) of cases observed in 2024 were avertable, while the reestablishment of IRS in late 2024 averted an additional increase of 7.0% (95% CI 3.8–10.2%) (Figure 2).

Notably, the impact of the interruption of malaria control and reintroduction of IRS varied across districts. Given its high population, estimates for Malabo were highly similar to those overall, with 29.8% of 2024 cases avertable (95% CI 13.6–43.3%) and the reintroduction of IRS averting a further increase of 7.9% (95% CI 3.8–11.7%). In Baney, while the increase in cases observed was not found to be statistically significant, reintroducing IRS in late 2024 was estimated to have averted an additional increase in cases of 5.9% (95% CI 3.0–8.4%). In Luba and Riaba, no statistically significant impact of the interruption or reestablishment of control activities was identified, although the mean estimates of avertable cases in Riaba (24.1% of cases reported in 2024) and cases averted by reestablishment of control in Luba (4.2% further increase in cases averted) were comparable in magnitude to higher population districts with statistically significant findings (Figure 2).

### Changes in prevalence

**Figure 3:**
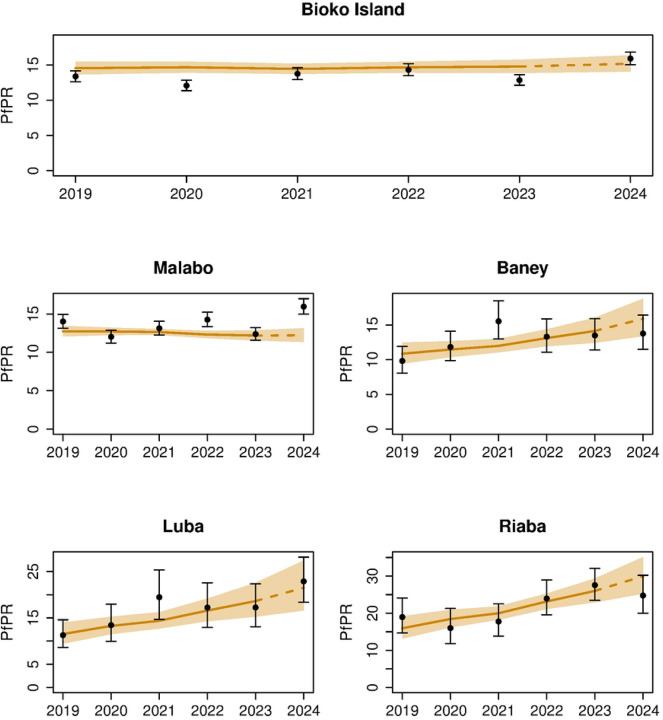
Estimated and observed *Pf*PR overall and by district from 2019–2024. Solid line and ribbon indicate model fit and 95% CI, and the dashed line corresponds the 2019–2023 trend extrapolated to 2024. Points and whiskers indicate observed *Pf*PR.

**Figure 4:**
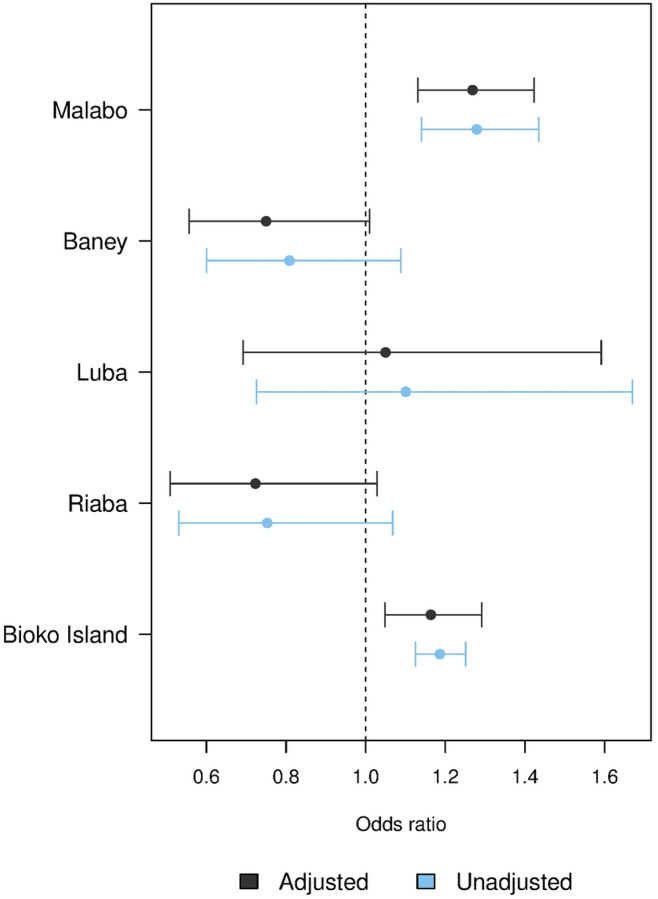
Adjusted and unadjusted odds ratios (OR) of *Pf*PR attributed to the interruption of malaria control activities in 2024, compared to 2019–2023 trend, by district, stratum and overall. Covariates used in adjustment were LLIN use, recent travel history, early house entry and SES quintile. Points show estimates, whiskers indicate the 95% CI, and the vertical dotted line corresponds to no effect (OR=1).

In the six years of MIS included in this analysis, 29,902 household surveys were conducted with 77,774 RDTs administered, and all covariates used in adjustment were available for the vast majority of these individuals (Table 1). Overall, there was a three percentage-point increase in *Pf*PR from 12.9% in 2023 to 15.9% in 2024, primarily driven by an increase in Malabo district (Figure 3). The overall increase corresponded to an adjusted odds ratio of 1.16 above the 2019–2023 trend (95% CI 1.05–1.29, unadjusted OR 1.19, 95% CI 1.12–1.25). However, at a district level the effect of service interruptions was statistically significant only in Malabo, where the increase mirrored the overall results (adjusted OR 1.27, 95% CI 1.13–1.42) (Figure 4). In Luba no significant increase beyond previous trends was identified (adjusted OR 1.05, 95% CI 0.69–1.59). Counterintuitively, Baney and Riaba registered lower *Pf*PR than expected from previous trends, although these were (narrowly) not statistically significant (adjusted OR 0.75 95% 0.56–1.01 and 0.72, 95% CI 0.51–1.03, respectively).

## Discussion

The results reported here demonstrate the speed with which gains from malaria control can be lost, but also that reintroduction of control can regain lost ground just as quickly. In a period of only one year without IRS on Bioko, cases rose by nearly 50%, while prevalence returned to levels last seen around 10 years ago [24]. Unsurprisingly, these changes were significant departures from the pre-interruption trends, adding to the already large body of evidence another example of resurgence caused by interruption to control operations [5]. Even on Bioko, this is not the first documented impact of funding interruptions. In 2019, an outbreak was registered in Riaba, at least partially caused by a delay in IRS implementation due to a lapse in funding [25]. However, the 2024 funding disruption was substantially longer and appears to have had more widespread effects, including in the urban portion of the island (principally, in Malabo district). More importantly, the data presented here demonstrate that while resurgences are rapid, they can also be quickly contained by the reintroduction of control activities. Despite substantial resurgence in reported cases, by the end of 2024 the reestablishment of control reduced monthly case counts to a level comparable to what would have been expected with no interruption.

Interestingly, analyses of cases and prevalence data did not entirely agree. Both analyses found a higher than otherwise expected burden in 2024 overall and for Malabo district, and no statistically significant change in Luba or Riaba. However, meaningful (although not statistically significant) decreases in prevalence compared to previous trends were found in Baney and Riaba, while analysis of confirmed cases showed a statistically significant decrease related to the re-establishment of IRS in Baney and a sizeable but non-significant increase attributable to the interruption in Riaba. In the case of Riaba, this apparent inconsistency was likely caused by a decreasing interannual trend identified in case data starting from 2023, which was not apparent in MIS data in large part due to their temporal sparsity. Similarly, the inconsistency in findings for Baney may stem from different long-term (interannual) trends identified: an increasing trend from MIS data, and a stable trend in case data.

It is also important to emphasize that the increase in burden reported here is not reflective of a full interruption of malaria control activities. Since the disruption lasted only one year, the impact on LLIN use was limited (Table 1). Similarly, temporary agreements with funders limited the impact that the disruption had on routine case management, for example avoiding stockouts of diagnostic tools and antimalarial treatments. Thus, the effects observed are primarily due to an interruption of IRS and would almost certainly be larger for a longer lasting or more complete interruption. A similarly rapid resurgence of cases following a cessation of IRS was observed in Uganda, although the interruption was longer and the effect was larger than that observed here [6]. The impact of such disruptions on prevalence is less well-studied, mostly due to the difficulty of collecting prevalence data during funding lapses. However, the overall increase in *Pf*PR reported here is broadly consistent with the effect size of IRS estimated from a historical analysis of Bioko Island MIS data [26], further supporting the interpretation that these changes are caused primarily by the interruption to IRS. More broadly, the effect sizes observed here may be particular to the context of Bioko, with its long history of intense control (principally, IRS).

The main weakness of this study is the assumption that, when extrapolated to 2024, observed pre-interruption trends provide a reasonable estimate of what would have occurred had activities not been interrupted. In particular, the opening of a new public hospital in 2024 is potentially problematic for the attribution of changes in reported cases to control interruptions. However, the rapid decline in cases after the reestablishment of IRS in late 2024, including those reported from the General Hospital of Sampaka, suggests that increases in confirmed cases are more likely to have been caused by the lack of IRS than changes in the health system. Similarly, the use of linear temporal trends for MIS analyses could obscure important pre-interruption variation, although this does not appear to be a major issue on visual inspection (Figure 3). Additionally, the small populations and of Luba and Riaba limit the interpretation of results for these districts. Despite these weaknesses, the overall consistency of results of case and prevalence analyses, and especially the impact on cases seen after the reestablishment of IRS in late 2024 provide relatively strong evidence that the increase in burden observed was caused primarily by the interruption of IRS.

## Conclusion

This study provides a clear and well-documented case of the impacts that interruptions to malaria control and reintroductions of control can have. Substantial increases in confirmed malaria cases and malaria prevalence were observed after less than one year with limited control. While this resurgence was rapid, it was also quickly contained and reversed by the reintroduction of control activities. These are important considerations for funders when taking decisions on reducing or re-allocating malaria control funding, as well as when responding to subsequent resurgences.

## Figures and Tables

**Table 1:** Characteristics of MIS participants and covariates. Columns labelled “Analytical Dataset” correspond to observations used in final (adjusted and unadjusted) analysis, with non-missing values for adjustment covariates. Note that individuals and households with unknown age or undefined SES quintile are excluded from the age and SES quintile breakdowns, respectively.

	All Data			Analytical Dataset					
	Households surveyed	Individuals tested	*Pf*PR	Households surveyed	Individuals tested	*Pf*PR	LLIN use	Early house entry	Travel history
**Year**									
2019	5,074	13,918	13.4 %	4,402	12,639	13.1 %	38.4 %	28.4 %	10.7 %
2020	4,963	12,738	12.1 %	3,971	11,236	11.7 %	37.2 %	22.2 %	1.9 %
2021	4,928	12,515	13.8 %	4,064	11,018	13.5 %	34.5 %	27.9 %	2.9 %
2022	5,026	12,337	14.3 %	4,141	10,769	13.9 %	36.1 %	32.3 %	6.3 %
2023	4,998	13,300	12.9 %	4,170	11,490	12.4 %	34.2 %	32.8 %	6.6 %
2024	4,913	12,966	15.9 %	4,105	11,381	15.2 %	29.0 %	29.3 %	5.1 %
**District**									
Malabo	22,690	59,342	13.6 %	18,556	52,189	13.2 %	35.2 %	28.0 %	5.9 %
Baney	3,709	10,260	12.9 %	3,178	9,053	12.6 %	31.3 %	34.6 %	5.2 %
Luba	2,150	4,671	16.4 %	1,904	4,202	16.0 %	47.8 %	23.1 %	2.5 %
Riaba	1,353	3,501	21.8 %	1,215	3,089	21.5 %	36.4 %	25.8 %	2.9 %
**SES quintile**									
Lowest	7,400	12,817	16.6 %	5,961	11,274	16.3 %	39.0 %	21.6 %	3.2 %
Second	6,017	14,848	16.0 %	5,053	13,115	15.5 %	42.5 %	25.8 %	4.0 %
Middle	5,495	15,537	14.6 %	4,662	13,683	14.1 %	39.9 %	27.1 %	4.8 %
Fourth	5,544	16,839	13.0 %	4,727	14,892	12.4 %	36.7 %	30.7 %	5.8 %
Highest	5,421	17,686	10.4 %	4,450	15,569	10.2 %	21.3 %	34.5 %	9.0 %
**Age**									
<5		10,777	8.2 %		9,643	8.0 %	41.2 %	44.1 %	3.1 %
5–14		23,214	17.1 %		20,220	16.7 %	35.6 %	33.7 %	3.0 %
15+		43,342	13.3 %		38,261	12.9 %	32.8 %	22.0 %	7.8 %
**Overall**	**29,902**	**77,774**	**13.7 %**	**24,853**	**68,533**	**13.3 %**	**34.9 %**	**28.8 %**	**5.7 %**

## Data Availability

All data and code used in this study are available at the GitHub repository https://github.com/galickda/bioko_interruption_2024

## References

[1] World Health Organization. World malaria report 2024: addressing inequity in the global malaria response. Geneva: World Health Organization; 2024.

[2] Bhatt S, Weiss DJ, Cameron E, Bisanzio D, Mappin B, Dalrymple U, The effect of malaria control on Plasmodium falciparum in Africa between 2000 and 2015. Nature. 2015 Oct;526(7572):207–11. 26375008 10.1038/nature15535 PMC4820050

[3] Feachem RGA, Chen I, Akbari O, Bertozzi-Villa A, Bhatt S, Binka F, Malaria eradication within a generation: ambitious, achievable, and necessary. The Lancet. 2019 Sept;394(10203):1056–112. 10.1016/S0140-6736(19)31139-0 31511196

[4] Lawal L, Buhari AO, Jaji TA, Alatare AS, Adeyemo AO, Olumoh AO, Lingering challenges in malaria elimination efforts in sub-Saharan Africa: Insights and potential solutions. Health Sci Rep. 2024;7(6):e2122. 38831778 10.1002/hsr2.2122 PMC11144596

[5] Cohen JM, Smith DL, Cotter C, Ward A, Yamey G, Sabot OJ, Malaria resurgence: a systematic review and assessment of its causes. Malar J. 2012 Apr 24;11:122. 22531245 10.1186/1475-2875-11-122 PMC3458906

[6] Namuganga JF, Epstein A, Nankabirwa JI, Mpimbaza A, Kiggundu M, Sserwanga A, The impact of stopping and starting indoor residual spraying on malaria burden in Uganda. Nat Commun. 2021 May 11;12(1):2635. 33976132 10.1038/s41467-021-22896-5 PMC8113470

[7] Janko MM, Recalde-Coronel GC, Damasceno CP, Salmón-Mulanovich G, Barbieri AF, Lescano AG, The impact of sustained malaria control in the Loreto region of Peru: a retrospective, observational, spatially-varying interrupted time series analysis of the PAMAFRO program. Lancet Reg Health – Am. 2023 Apr 1;20. 10.1016/j.lana.2023.100477 PMC10036736 36970494

[8] Dzianach PA, Rumisha SF, Lubinda J, Saddler A, van den Berg M, Gelaw YA, Evaluating COVID-19-Related Disruptions to Effective Malaria Case Management in 2020–2021 and Its Potential Effects on Malaria Burden in Sub-Saharan Africa. Trop Med Infect Dis. 2023 Apr;8(4):216. 37104342 10.3390/tropicalmed8040216 PMC10143572

[9] Winskill P, Slater HC, Griffin JT, Ghani AC, Walker PGT. The US President’s Malaria Initiative, Plasmodium falciparum transmission and mortality: A modelling study. PLOS Med. 2017 Nov 21;14(11):e1002448. 29161259 10.1371/journal.pmed.1002448 PMC5697814

[10] Yukich JO, Chitnis N. Modelling the implications of stopping vector control for malaria control and elimination. Malar J. 2017 Oct 13;16(1):411. 29029609 10.1186/s12936-017-2051-1 PMC5640964

[11] Sherrard-Smith E, Hogan AB, Hamlet A, Watson OJ, Whittaker C, Winskill P, The potential public health consequences of COVID-19 on malaria in Africa. Nat Med. 2020 Sept;26(9):1411–6. 32770167 10.1038/s41591-020-1025-y PMC7613562

[12] García GA, Hergott DEB, Phiri WP, Perry M, Smith J, Osa Nfumu JO, Mapping and enumerating houses and households to support malaria control interventions on Bioko Island. Malar J. 2019 Aug 22;18(1):283. 31438979 10.1186/s12936-019-2920-x PMC6704714

[13] Fries B, Guerra CA, García GA, Wu SL, Smith JM, Oyono JNM, Measuring the accuracy of gridded human population density surfaces: A case study in Bioko Island, Equatorial Guinea. PLOS ONE. 2021 Sept 1;16(9):e0248646. 34469444 10.1371/journal.pone.0248646 PMC8409626

[14] Kleinschmidt I, Schwabe C, Benavente L, Torrez M, Ridl FC, Segura JL, Marked Increase in Child Survival after Four Years of Intensive Malaria Control. Am J Trop Med Hyg. 2009 June;80(6):882–8. 19478243 PMC3748782

[15] Overgaard HJ, Reddy VP, Abaga S, Matias A, Reddy MR, Kulkarni V, Malaria transmission after five years of vector control on Bioko Island, Equatorial Guinea. Parasit Vectors. 2012 Nov 12;5(1):253. 23146423 10.1186/1756-3305-5-253 PMC3533880

[16] García GA, Galick DS, Smith JM, Iyanga MM, Rivas MR, Eyono JNM, The challenge of improving long-lasting insecticidal nets coverage on Bioko Island: using data to adapt distribution strategies. Malar J. 2024 Oct 29;23(1):324. 39472916 10.1186/s12936-024-05139-y PMC11523664

[17] García GA, Hergott DEB, Galick DS, Donfack OT, Motobe Vaz L, Nze Nchama LO, Testing indoor residual spraying coverage targets for malaria control, Bioko, Equatorial Guinea. Bull World Health Organ. 2025 June 1;103(6):392–402. 40511394 10.2471/BLT.24.292505 PMC12161160

[18] Cook J, Hergott D, Phiri W, Rivas MR, Bradley J, Segura L, Trends in parasite prevalence following 13 years of malaria interventions on Bioko island, Equatorial Guinea: 2004–2016. Malar J. 2018 Feb 5;17(1):62. 29402288 10.1186/s12936-018-2213-9 PMC5799938

[19] Roll Back Malaria. Malaria Indicator Survey Toolkit [Internet]. Available from: https://www.malariasurveys.org/toolkit.cfm

[20] Citron DT, Guerra CA, García GA, Wu SL, Battle KE, Gibson HS, Quantifying malaria acquired during travel and its role in malaria elimination on Bioko Island. Malar J. 2021 Aug 30;20(1):359. 34461902 10.1186/s12936-021-03893-x PMC8404405

[21] García GA, Atkinson B, Donfack OT, Hilton ER, Smith JM, Eyono JNM, Real-time, spatial decision support to optimize malaria vector control: The case of indoor residual spraying on Bioko Island, Equatorial Guinea. PLOS Digit Health. 2022 May 12;1(5):e0000025. 36812503 10.1371/journal.pdig.0000025 PMC9931250

[22] Wood SN. Generalized Additive Models: An Introduction with R. 2nd ed. Chapman and Hall/CRC; 2017.

[23] Hergott DEB. Impact of six-month COVID-19 travel moratorium on Plasmodium falciparum prevalence on Bioko Island, Equatorial Guinea. Nat Commun. 2024; 10.1038/s41467-024-52638-2 PMC11436818 39333562

[24] National Malaria Control Program of Equatorial Guinea, MCD Global Health. Bioko Island Malaria Indicator Survey 2024. 2025.

[25] Guerra CA, Fuseini G, Donfack OT, Smith JM, Ondo Mifumu TA, Akadiri G, Malaria outbreak in Riaba district, Bioko Island: lessons learned. Malar J. 2020 Aug 3;19(1):277. 32746919 10.1186/s12936-020-03347-w PMC7398070

[26] Galick DS, Vaz LM, Ondo L, Iyanga MM, Bikie FEE, Avue RMN, Reconsidering indoor residual spraying coverage targets: A retrospective analysis of high-resolution programmatic malaria control data. Proc Natl Acad Sci. 2025 Apr 22;122(16):e2421531122. 40228135 10.1073/pnas.2421531122 PMC12037036

